# GLAUCOMA, CATARACT, AND EMOTIONAL DISTRESS IN NEW ENGLAND: FINDINGS FROM THE HEALTHY AGING DATA REPORTS

**DOI:** 10.1093/geroni/igac059.2319

**Published:** 2022-12-20

**Authors:** Shu Xu, Chae Man Lee, YanJhu Su, Taylor Jansen, Nina Silverstein, Beth Dugan

**Affiliations:** University of Massachusetts Boston, Boston, Massachusetts, United States; University of Massachusetts Boston, Boston, Massachusetts, United States; University of Massachusetts Boston, Boston, Massachusetts, United States; University of Massachusetts Boston, Boston, Massachusetts, United States; University of Massachusetts Boston, Boston, Massachusetts, United States; University of Massachusetts Boston, Boston, Massachusetts, United States

## Abstract

Glaucoma and Cataract are two common leading causes of vision loss and major sources of disability and mental issues for older adults, but few studies investigated glaucoma and cataract among older adults from a community-level perspective. This study describes the rates of glaucoma and cataract among older adults 65+ in New England and examined the association of glaucoma and cataract and emotional distress (depression and anxiety) of older adults at the community level across 794 towns/cities (MA (351), NH (235), RI (39), and CT (169)) in New England. Data sources used to calculate rates of communities were: the American Community Survey (2014-2018 RI and CT, 2012-2016 MA and NH) and the Medicare Current Beneficiary Summary File (2016-2017 RI, 2015 MA and NH, 2016-2017 CT). Small area estimation techniques were used to calculate age-sex adjusted community rates for more than 150 health indicators (https://healthyagingdatareports.org/). Results showed CT had the highest rates of glaucoma (28.3%), RI had the highest rates of cataract (67.5%), and NH had the lowest rates of glaucoma and cataract (22.9% and 61.2%, respectively). After adjusting for age, gender, race, marital status, education, income, chronic diseases, communities with higher rates of glaucoma or cataract were significantly associated with higher rates of emotional distress compared to those with lower prevalence (glaucoma: β=0.06, p< 0.01, for depression, β=0.09, p< 0.001 for anxiety; cataract: β=0.08, p< 0.01 for depression, β=0.11, p< 0.01 for anxiety). Findings help practitioners and policymakers to allocate emotional support services to areas of high need due to eye diseases.

